# Tuning Compressive Young’s Moduli and Antibacterial Activities of Alginate/Poly(*N*-isopropylacrylamide) Hydrogels with Laponite Layers and Cerium Ions

**DOI:** 10.1021/acsomega.2c03937

**Published:** 2022-10-03

**Authors:** Çiçek Endeşav, Bestenur Yalçın, Ceyda Şimşek, Candan Erbil

**Affiliations:** † Faculty of Science and Letters, Department of Chemistry, Istanbul Technical University, Istanbul, TR34469, Turkey; ‡ Department of Medical Laboratory Techniques Istanbul, Bahcesehir University Vocational School of Health Services, Istanbul, TR34353, Turkey

## Abstract

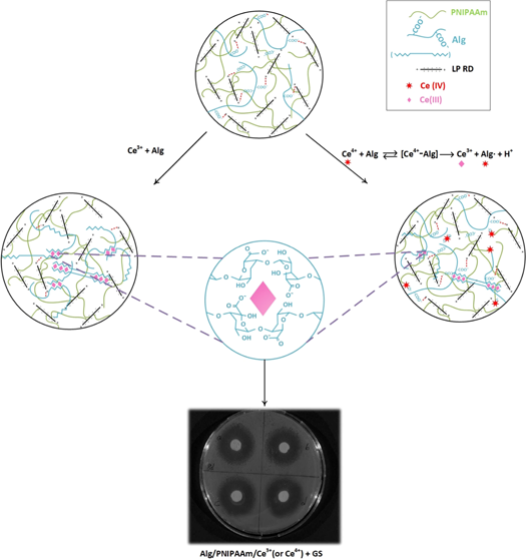

Hybrid hydrogels containing alginate (Alg) and poly(*N*-isopropylacrylamide) (PNIPAAm) chains as natural and synthetic components, respectively, were crosslinked using double and triple pairs of the crosslinkers Ce^3+^/Ce^4+^, laponite (LP) RD, and *N*,*N*’-methylenebisacrylamide (BIS). (Alg/PNIPAAm)-Ce^3+^ and (Alg/PNIPAAm-PNIPAAm)-Ce^3+^ double- and triple-network structures were prepared using multivalent cerium ions (Ce^3+^), multifunctional laponite layers (L), and/or neutral tetrafunctonal BIS molecules (B). Compressive Young’s moduli, E, were tuned by the type/concentration of crosslinkers and crosslinking procedures and the concentration of Alg chains. The antibacterial activity of positively charged ions and molecules is due to the electrostatic attraction with the negatively charged bacterial cell walls. In the current study, we report the antibacterial activity on *Escherichia coli* of Ce^3+^ ions in the absence and presence of gentamicin sulfate (GS) for double and triple networks. Nonbacterial areas, which are called inhibition zones, around the disks, and compressive E moduli of the single and double PNIPAAm and Alg/PNIPAAm networks crosslinked by LP RD and containing Ce^3+^/Ce^4+^ions in free and ionically bonded states, respectively, were higher than those of the ones crosslinked with BIS. Moreover, BIS- and LP RD-crosslinked single PNIPAAm hydrogels displayed larger inhibition zones than those of Alg/PNIPAAm hybrids, supporting the antibacterial activity of free Ce^3+^/Ce^4+^ ions diffused together with GS molecules. On the other hand, antibacterial activities of GS + Ce^3+^-loaded triple networks were much lower than those of their double counterparts because the increase in the structural complexity reduced the co-emission of antibacterial agents.

## Introduction

Hydrogels having superior mechanical properties, optical transparency, ionic conductivity, stimulus responsiveness, and biocompatibility are used as soft robots, and actuators, sensors, communicators, and power sources can be prepared using these hydrogel-based soft robots.^1−5^ Biopolymers such as cellulose, alginate (Alg), and gelatin have also been used to produce biodegradable materials for soft robotics.^6^

Hybrid hydrogels contain synthetic and natural components in the form of linear and/or crosslinked polymer chains or crosslinkers. Carbohydrate polymers are a specific class of natural and biological polymers, and cellulose, starch, dextrins, chitosan, hyaluronic acid, carrageenan, and Alg are among their most common examples found in nature. They are used in biomedical applications such as wound dressing and drug delivery due to the biocompatibility and similarity of their swollen hydrogels to living tissues.^7,8^

Alginate, which is an example of carbohydrate polymers and derived from brown seaweeds, is a mixed salt containing sodium and/or potassium, calcium, and magnesium salts of water-insoluble alginic acid. The distribution of homogeneous and heterogeneous mannuronate (M) and guluronate (G) units, such as GG, MM, GM, and MG, G:M proportions, and block lengths on linear alginate chains affect their gel formation ability with metal ions and thus the mechanical properties of the materials containing them.^9,10^ Zhou and Chen et al. reported the results of tensile tests and antibacterial activities of Alg/PAAm (polyacrylamide) hybrid hydrogels containing various multivalent ions as ionic crosslinkers.^11,12^

Poly(*N*-isopropylacryamide) crosslinked chemically and/or physically is the most studied member of thermosensitive hydrogels since its volume phase transition temperature (VPTT) is close to the human body temperature (32–34 °C).^13,14^ Zhou and Chen et al. also synthesized tough Alg/PNIPAAm hybrid hydrogels of Alg and PNIPAAm with ionic and chemical crosslinking using Al^3+^and *N*,*N’*-methylenebisacrylamide (BIS), respectively, by a double network procedure.^15^ Haraguchi reported that the mechanical properties, swelling/deswelling processes, and homogeneity of PNIPAAm hydrogels were markedly improved by clay layers acting as inorganic multicrosslinkers through ionic and/or polar interactions, compared to chemical crosslinking by divinyl crosslinkers.^16,17^ Ye et al. reported the preparation of pH- and temperature-sensitive hybrid PNIPAAm hydrogels composed of polyanionic Alg and laponite-XLG (synthetic hectorite) as a natural pH-sensitive component and multicrosslinker, respectively.^18^ In our previous studies, the effects of various initiator–activator pairs on the mechanical properties of both PNIPAAm-montmorillonite (MMT) and PNIPAAm-laponite RD hydrogels have been demonstrated.^19−21^

Cerium salts may exist as Ce^3+^ and Ce^4+^ ions that complex with hydroxyl ions in the aqueous solutions, and depending on the experimental conditions, they can cycle between these two oxidation states.^22,23^ The surfaces of cerium dioxide (CeO_2_) nanoparticles also contain both the oxidation states Ce^3+^ and Ce^4+^, and, the ratios of Ce^3+^:Ce^4+^, which are dependent on the synthesis conditions, have an important role in the biological activity.^24,25^ Thill et al. reported that CeO_2_ nanoparticle dispersions in water are positively charged at neutral pH, and thus, they exhibited strong electrostatic attraction toward outer membranes of Gram-negative bacteria.^26^

Although many results report the synergistic effect of cerium(III) nitrate in the composition of silver sulfadiazine cream in the treatment of deep burns, the mechanism of this action is unknown. According to some authors, with cerium nitrate added to the silver sulfadiazine cream, the crust formed on/around the wound increases its healing rate and decreases infection risk since there is no gap between the edges of the wound and the crust.^27,28^ Moreover, Ce^3+^ behaves chemically similar to Ca^2+^ due to the size and preferences toward donor atoms. Hence, it has the potential to replace calcium in many biomolecules. Yousheng et al. have investigated the antibacterial activity of Ce^3+^-MMT and observed the lower sensitivity of the Gram-negative bacterium *Escherichia coli* toward Ce^3+^-MMT compared to that of Gram-positive bacteria due to the complex structure of its outer membrane.^29^

PNIPAAm-based single, double, and triple hybrid hydrogels were synthesized using cerium salts (Ce^3+^/Ce^4+^), LP RD, and BIS as inorganic and organic crosslinkers and alginate chains as natural and ionic/hydrophilic components. The novelty of this study is tuning the compression moduli and antibacterial activities by changing crosslinker combinations. Gentamicin sulfate (GS) as an antibacterial drug was used, along with cerium(III) nitrate hexahydrate and cerium(IV) ammonium nitrate salts, against *E. Coli*, for qualitative disk diffusion tests.

## Materials and Methods

### Materials

*N*-isopropylacrylamide (NIPAAm; Aldrich, St. Louis) as a monomer, *N*,*N*,*N^’^*,*N*^’^- tetramethyl ethylenediamine (TEMED; Sigma-Aldrich, Steinheim, Germany) as an activator, and potassium persulfate (KPS; Merck, Darmstadt, Germany) as an initiator were used without further purification. Sodium alginate (Alg; Protonal LFR 5/60) was gifted by FMC Company and used as received. The range of the G/M ratio of this low-molecular weight (20–60 kDa) product is 65-75/35-25. *N*,*N’*-methylenebisacrylamide (BIS; Merck, Darmstadt, Germany) and laponite RD (LP RD; Na^+0.7^[(Si_8_Mg_5.5_Li_0.3_) O_20_(OH)_4_]^−0.7^; Rockwood Additive Limited, U.K.) were chosen as organic tetrafunctional and inorganic multifunctional crosslinkers, respectively.

Distilled–deionized water (DDW; pH ≈ 5.5), supplied using a Millipore RiOs-DI 3 UV water purification system, was used in experiments. Phosphate buffer solution (PBS; pH ≈ 7.4) for antibacterial activity and mechanical tests was prepared using NaCl (8 g/L), KCl (0.2 g/L), Na_2_HPO4.2H2O (1.78 g/L), and KH_2_PO4 (0.24 g/L) (Merck, Darmstadt, Germany). Gentamicin sulfate (GS; Sigma-Aldrich, Steinheim, Germany), which is an effective antibacterial agent for especially Gram-negative bacterial infections, was used as a model drug. Cerium(IV) ammonium nitrate ((NH_4_)_2_ Ce (NO_3_)_6_; Ce^4+^) and cerium(III) nitrate hexahydrate (Ce(NO_3_)_3_·6H_2_O; Ce^3+^; Aldrich, St. Louis) were used for ionic gelation and antibacterial activity.

### Synthesis of Single/Double/Triple Hybrid Hydrogels

(i) ***PNIPAAm hydrogels B0 and L0*** crosslinked with BIS (2.50 × 10^–2^ mol/L) and LP RD (10% by weight of the NIPAAm (1.0 mol/L) used) were prepared using KPS (5.00 × 10^–3^ mol/L)/TEMED (1.50 × 10^–2^ mol/L) as an initiator/activator pair at 25 °C in DDW. After 2w, chosen as the reaction time, all cylindrical samples synthesized in glass tubes were cut into disks after the tubes were broken off and then purified by immersing into excess DDW for 1w to remove unreacted constituents.
(ii) ***Alg/PNIPAAm hybrid hydrogels B2/B4/B6 and L2/L4/L6*** contained three different concentrations of Alg (2.0, 4.0, and 6.0%; w/w) and are crosslinked with two different crosslinkers (BIS; 2.50 × 10^–2^ mol/L and LP RD; 10 wt %). The amounts of both Alg and LP RD (in wt %) were calculated with regard to the concentration of NIPAAm (1.0 mol/L) used. NIPAAm, BIS (or LP RD), and activator TEMED were dissolved in aqueous alginate solutions. The pre-gel mixtures poured into glass tubes were gelated using KPS as a free radical initiator under a nitrogen atmosphere at 25 °C. After 2w, chosen as the reaction time, all cylindrical samples synthesized in glass tubes were cut into disks after the tubes were broken off and then purified by immersing into excess DDW for 1w to remove unreacted constituents.
(iii) ***Ce^3+^-Alg/PNIPAAm hybrid hydrogels B2(/B4/B6)-3a (or b)/s and L2(/L4/L6)-3a (or b)/s*** were prepared by a two-step method. All cylindrical hydrogels synthesized in glass tubes during the first step were cut into disks with a height of 2 mm, after the tubes were broken off, and purified by immersing into DDW to remove unreacted species. In the second step called external gelation, Alg/PNIPAAm disks were immersed into the aqueous solutions (s) containing Ce(NO_3_)_3_·6H_2_O for 2 days. For Ce^3+^ crosslinking, i.e., double-network formation, two different concentrations were used: 5.0 × 10^–3^ mol/L (a) and 4.0 × 10^–2^ mol/L (b). Afterward, the disc-shaped specimens were washed with DDW to remove the free cerium ions on the outer surfaces.
(iv) ***Ce^4+^-Alg/PNIPAAm hybrid hydrogels B2(/B4/B6)-4a (or b)/t and L2(/L4/L6)-4a (or b)/t*** were synthesized in glass tubes. After taking out from the tubes by breaking, the cylindrical hydrogels were transferred into wider glass tubes (t) without purification, and the aqueous solutions of cerium(IV) ammonium nitrate were injected around them. The formation of double networks by both covalent and ionic bonds was possible during this second step. In the case of crosslinking with Ce^4+^, two different concentrations were used (5.00 × 10^–3^ mol/L (a) and 4.00 × 10^–2^ mol/L (b)) just as with Ce^3+^. After 2 days, the disc-shaped specimens cut from the cylindrical hydrogels were washed with DDW to remove the free cerium ions on their outer surfaces and the other unreacted species.
(v) ***L0/B0 and L2/B0 double networks crosslinked using BIS molecules and LP RD layers and L0/B0-3a/s and L2/B0-3a/s triple networks prepared using the external gelation method with Ce^3+^*** ions were synthesized in three steps. After the syntheses as described in (i) of single networks of hybrid hydrogels L0 and L2 were completed, the pre-gel mixture of the second network B0 was added around the first hydrogel (L0 or L2) in the glass tube with a syringe. The tubes were kept in a refrigerator so that the hydrogels L0 and L2 could absorb the added solution of B0 before the gelation processes for ***L0/B0 and L2/B0 double networks*** at 25 °C under a nitrogen atmosphere. After 2 weeks, chosen as the reaction time, the cylindrical double networks were cut into disks and then purified by immersing into DDW to remove unreacted constituents. For triple network formation, the disks L0/B0 and L2/B0 were immersed into an aqueous solution containing 5.00 × 10^–3^ mol/L Ce(NO_3_)_3_·6H_2_O (3a/s) for 2 days. Afterward, the disc-shaped specimens were washed with DDW to remove the free Ce^3+^ ions on the outer surfaces.

The crosslinker type in the structures of the Alg/PNIPAAm hydrogels was expressed using simple notations such as L (for LP RD), B (for BIS), 3 (for Ce^3+^), and 4 (for Ce^4+^) in all the figures and tables, while alginate contents (in wt %; in the feed) in the PNIPAAm-based hybrid hydrogels were named as 0, 2, 4, and 6. The letters “a” and “b” next to the numbers “3” and “4” in the hydrogel labelings refer to two different concentrations of aqueous solutions of both cerium(III) nitrate hexahydrate and cerium(IV) ammonium nitrate, and “s” and “t” indicate external gelation of the hydrogel disks inserted into Ce^3+^ aqueous solutions and reaction/external gelation of the cylindrical hydrogels placed in Ce^4+^solutions in glass tubes, respectively.

### Uniaxial Compression Tests

Disc-shaped samples of hybrid hydrogels were subjected to the static uniaxial compression test performed using a single-column mechanical tester (Hounsfield HK-5S Model) equipped with a load cell of 5.0 N. First, their heights and diameters were measured, after the hydrogel disks achieved swelling equilibria at 25° and 37 °C in DDW and PBS. Then, the compressive force was applied at a rate of 10 mm/min from 0.0 to 5.0 N. During all the measurements, the disks were set on the lower plate and compressed with the upper plate, which was connected to a load cell. According to the following equation, the value of compressive Young’s modulus, E, for each of the hydrogel disks was obtained from the slope of the linear part (between 0 and 10% strain) of the compressive stress–strain curve drawn by the recorded values of force and displacement
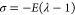
λ is obtained from the ratio of the deformed height (*h*) of the disk to the initial height (*h*_o_), λ = *h*/*h*_o_. σ refers to compressive stress, defined as the measured load (*F*) divided by the undeformed cross-sectional area (*A*_o_).

### Qualitative Antibacterial Activity Tests

Qualitative antibacterial tests of double/triple hybrid hydrogels based on Alg/PNIPAAm single networks were performed using the disk diffusion method. Hydrogel disks were dried to constant weight before inhibition zone tests and then immersed into PBS at pH 7.4 and PBS + GS solutions containing 0.2 wt % of GS for 48 h. During these periods, GS molecules and PBS diffused into the single/double/triple networks and the systems reached equilibrium states. In the following process, all the hybrid hydrogels in PBS or PBS + GS solutions were kept in a water bath at 37 °C for 12 h. After that, the hydrogel disks with approximately equal heights were placed on tryptic soyagar (TSA) plates inoculated with *E. coli* bacteria and incubated at 37 °C overnight. The inoculum concentration was 1.0 × 10^6^ colony forming units per 500 μL. All measurements were performed under sterile conditions. After the incubation time, inhibition zones were monitored using a BIORAD ChemiDoc MP imaging system with an epi-white gray scale.

Control tests were carried out using both the paper disks impregnated with the solutions containing Ce^3+^/Ce^4+^ ions and Alg/PNIPAAm hydrogel disks crosslinked with Ce^3+^/Ce^4+^ ions without GS molecules. The procedure applied to the GS-unloaded samples was the same as that for GS-loaded disks of the Alg/PNIPAAm double/triple networks crosslinked with Ce^3+^/Ce^4+^ ions after LP RD layers and/or BIS molecules. Inhibition zone tests for each of them were repeated in triplicate.

## Results and Discussion

The reason for choosing NIPAAm as the main monomer is the VPTT of its homopolymer, which is close to the human body temperature. This increases usage possibility in biomedical applications. On the other hand, alginate biopolymers are exploited in wound dressing materials because their hydrophilic nature provides a moist environment to enhance the healing rate of wounds.^9,30^ Podstawczyk et al. evaluated the material and biological properties of three-dimensional (3D) and four-dimensional (4D) thermoinks composed of Alg chains, laponite layers (or BIS molecules), and PNIPAAm network, for wound healing applications and hydrogel actuators.^31,32^ Since Alg chains, LP RD layers, and Ce^3+^/Ce^4+^ ions are expected to impart hydrophilicity, elasticity, and antibacterial activity to the temperature-sensitive PNIPAAm hydrogel, the hydrogels synthesized in this study may also have the potential to be used as wound dressing. At this point, the following question could be asked: why were the E moduli and inhibition zone areas of hybrid PNIPAAm hydrogels affected by crosslinker types and combinations?

**Scheme 1:**
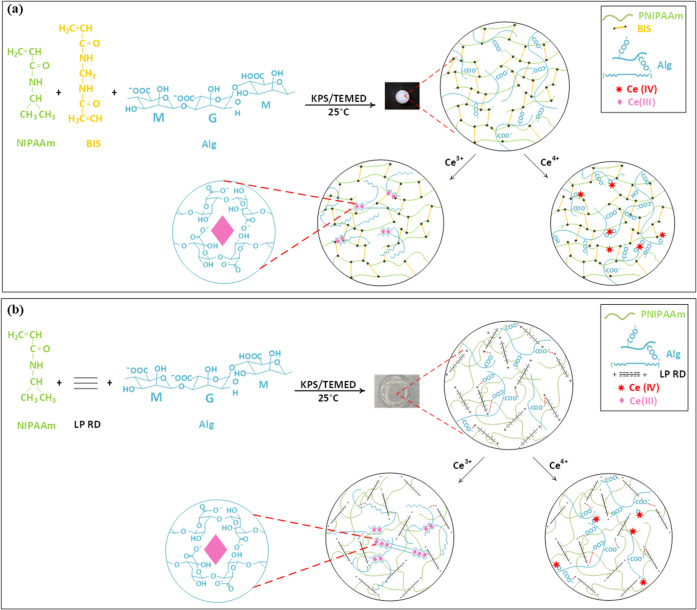
Two-Step Method for the Synthesis of Double Networks: PNIPAAm (B)/Alg (a) and PNIPAAm (L)/Alg (b) Hydrogels and their Second Crosslinks Formed by Ce^3+^/Ce^4+^ Ions

The compression mechanical properties and antibacterial activities of the single, double, and triple networks of Alg/PNIPAAm hydrogels were tuned using three different types of crosslinkers. Scheme 1 describes the PNIPAAm-based hydrogel formations via free radical solution polymerization with BIS molecules (a) or laponite layers (b) as crosslinkers, followed by external gelation with cerium ions. PNIPAAm and Alg/PNIPAAm hydrogels crosslinked with LP RD (L0/L2/L4/L6) can be considered comb-type hydrogels that the laponite layers loaded with negative charges behave like backbones decorated with many PNIPAAm brushes.^16,21^ The brushes initiated with the KPS/TEMED redox pair are connected onto the charged layers by ionic interactions. Some of the growing PNIPAAm brushes form bridges between different laponite layers, called multicrosslinkers. Furthermore, some −COO^–^ groups on the alginate chains present during the polymerization reactions can also interact electrostatically with the positive ends of the laponite layers.

**Figure 1:**
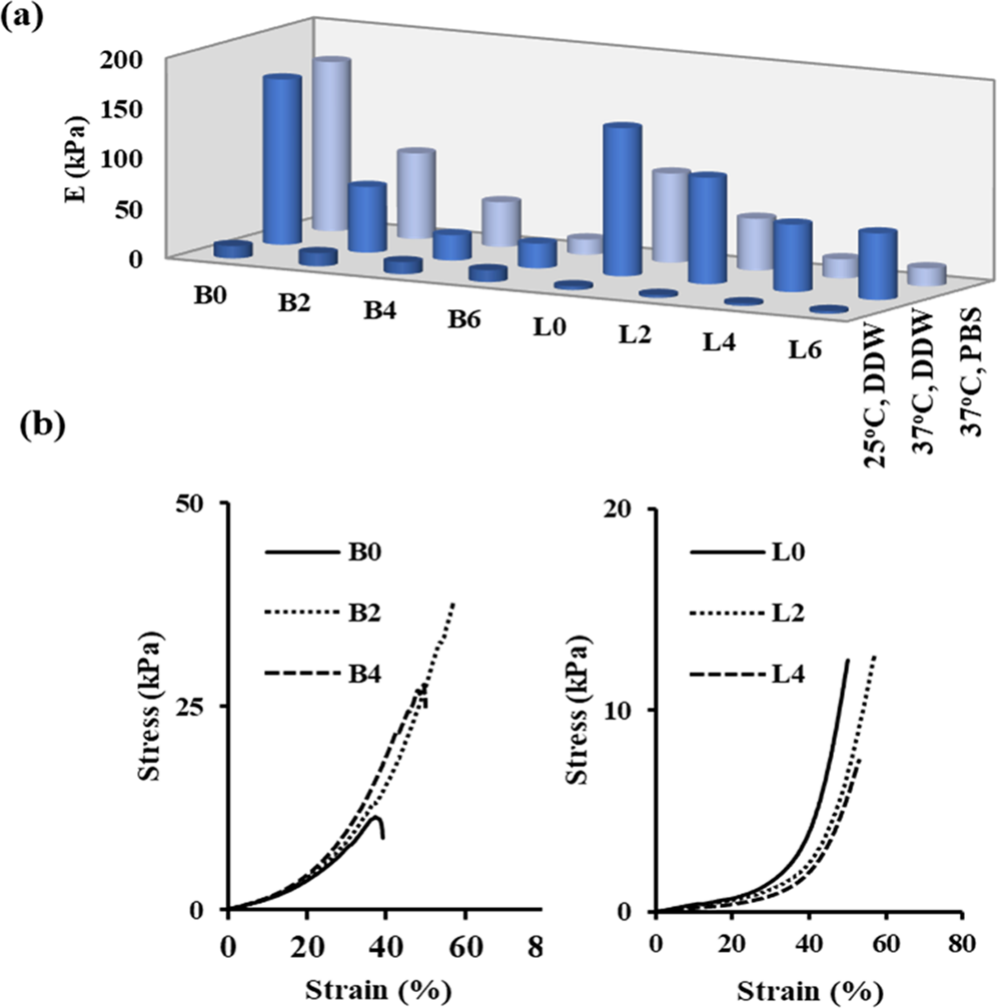
For PNIPAAm and Alg/PNIPAAm hydrogels crosslinked with BIS and LP RD, (a) compressive Young’s moduli at 25 and 37 °C in DDW (pH 5.5) and PBS (pH 7.4), and (b) compressive stress–strain curves at 25 °C in pH 5.5 DDW.

The fracture toughness of L-series hydrogels is higher than that of B-series (B0/B2/B4/B6) due to different energy dissipation mechanisms.^33−35^ If PNIPAAm chains between laponite layers are broken under compressive stress, their energies can be transferred to any others of the many chains as a consequence of the multifunctional inorganic crosslinker LP RD. On the other hand, PNIPAAm and Alg/PNIPAAm hydrogels crosslinked chemically with neutral and tetrafunctional BIS molecules, i.e., B-series, have more brittleness compared to that of L-series hydrogels due to static and short crosslinks between PNIPAAm chains (Figure 1).

**Figure 2:**
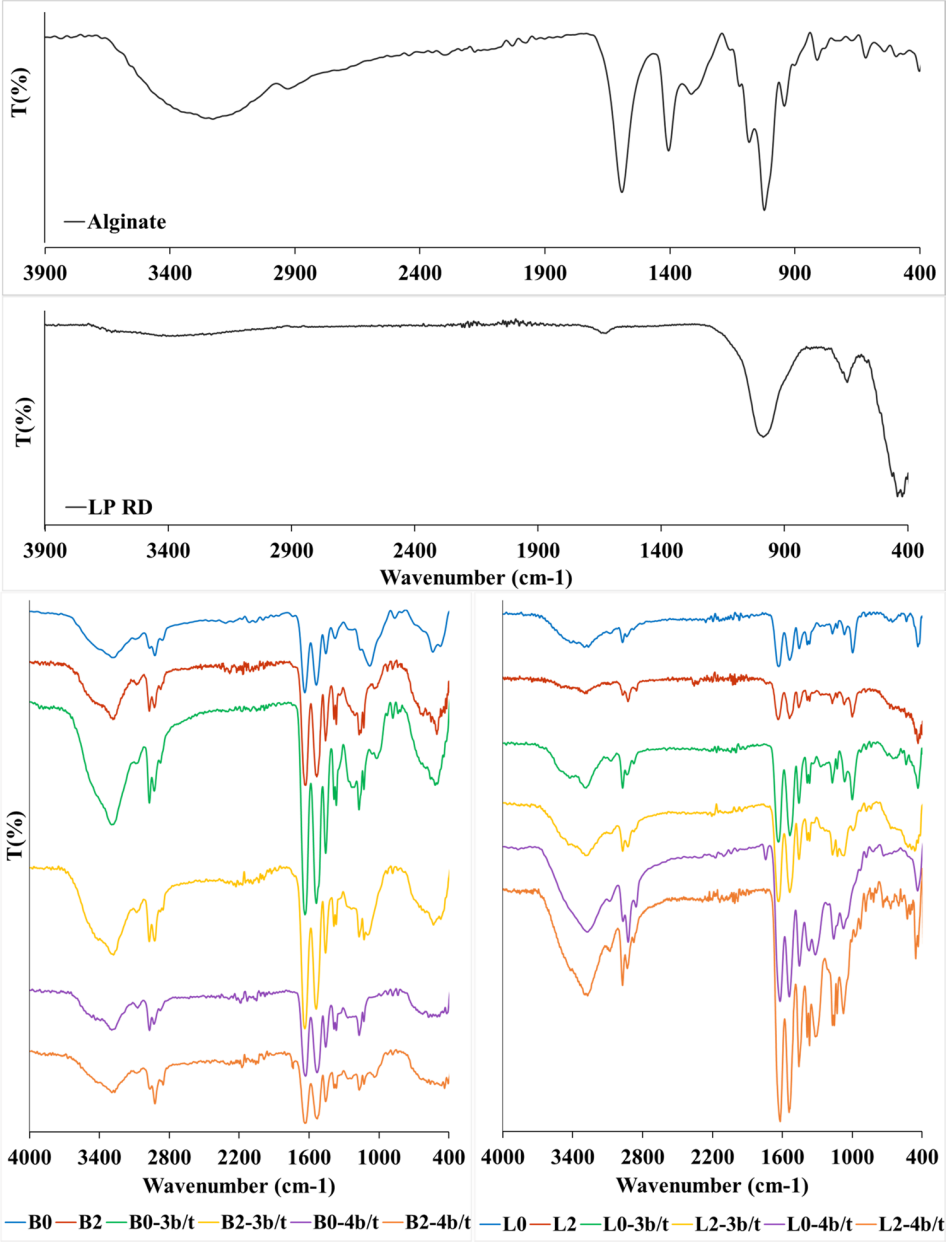
FTIR spectra of Alg, LP RD, and single/double networks of the PNIPAAm and Alg/PNIPAAm hydrogels crosslinked with BIS molecules, LP RD layers, and Ce^3+^/Ce^4+^ ions.

The Fourier transform infrared (FTIR) analysis was performed using a Bruker Alpha II model spectrometer, to evaluate the structures of Alg/PNIPAAm hybrid hydrogels crosslinked with BIS molecules, LP RD layers, and Ce^3+^/Ce^4+^ ions by chemical and physical interactions and external gelation, respectively. The FTIR spectra of Alg, LP RD, and single/double networks of the PNIPAAm-based hydrogels are presented in Figure 2.

The absorption bands at 1660 and 1540 cm^–1^ for C=O stretching and N–H bending of secondary amides, respectively, along with −CH vibration peak(s) at 1370–1385 cm^–1^ of the isopropyl group show the presence of PNIPAAm chains in the structures of hybrid hydrogels formed by free radical solution polymerizations.^36,37^ In the FTIR spectrum of Alg chains, the peaks at 1425 and 1625 cm^–1^ are ascribed to the symmetric and asymmetric stretching vibrations of −COO^–^ groups, respectively, while the stretching vibrations of hydroxyl groups of mannuronic and gluronic acids occur around 3425 cm^–1^. The minor peak at 1045 cm^–1^ and shoulder at 1740 cm^–1^ can be assigned to stretching vibrations of the C–O bond and −COOH group, respectively.^38−40^

The FTIR spectrum of LP RD shows a broad peak centered at 3400 cm^–1^ due to the O–H stretching vibrations of −SiOH groups and interlayer water molecules. The band at 990 cm^–1^ is due to the Si–O stretching vibration, while the peaks at 650 and 430 cm^–1^ correspond to Mg–O–Mg and Si–O–Mg bending, respectively.^2,41^

The Ce–O stretching peak at around 600 cm^–1^ and N–O peaks at 1330 and 1040 cm^–1^ reveal the presence of Ce ions and nitrate groups, respectively, and together with the peaks of LP RD indicated above support the presence of the inorganic crosslinkers in the structures of single and double networks of PNIPAAm-based hybrid hydrogels.^42^

FTIR spectra of B2-3b/t and L2-3b/t samples have nearly 90% similarity according to the results obtained twice, although both crosslinkers and transparency are different (Scheme 1). This structural characterization result supports the formation of two-stage (Alg/PNIPAAm)-Ce^3+^ double networks.

### Uniaxial Compression Tests

The importance of this study is to explain the relations between compressive Young’s moduli and antibacterial activities and compositions and molecular structures of the hybrid PNIPAAm hydrogels, based on the types and number of crosslinkers, and to observe the effects of solvent composition and temperature on mechanical properties. If the materials synthesized here are expected to have the potential to be used for biological applications such as drug delivery and wound dressings, they should also have good strength and elasticity along with biocompatibility and biodegradability. Therefore, the static uniaxial compression tests were performed for all the single, double, and triple networks equilibrated under both physiological conditions (i.e., at 37 °C in pH 7.4 PBS) and DDW at two different temperatures (25 and 37 °C).

**Scheme 2:**
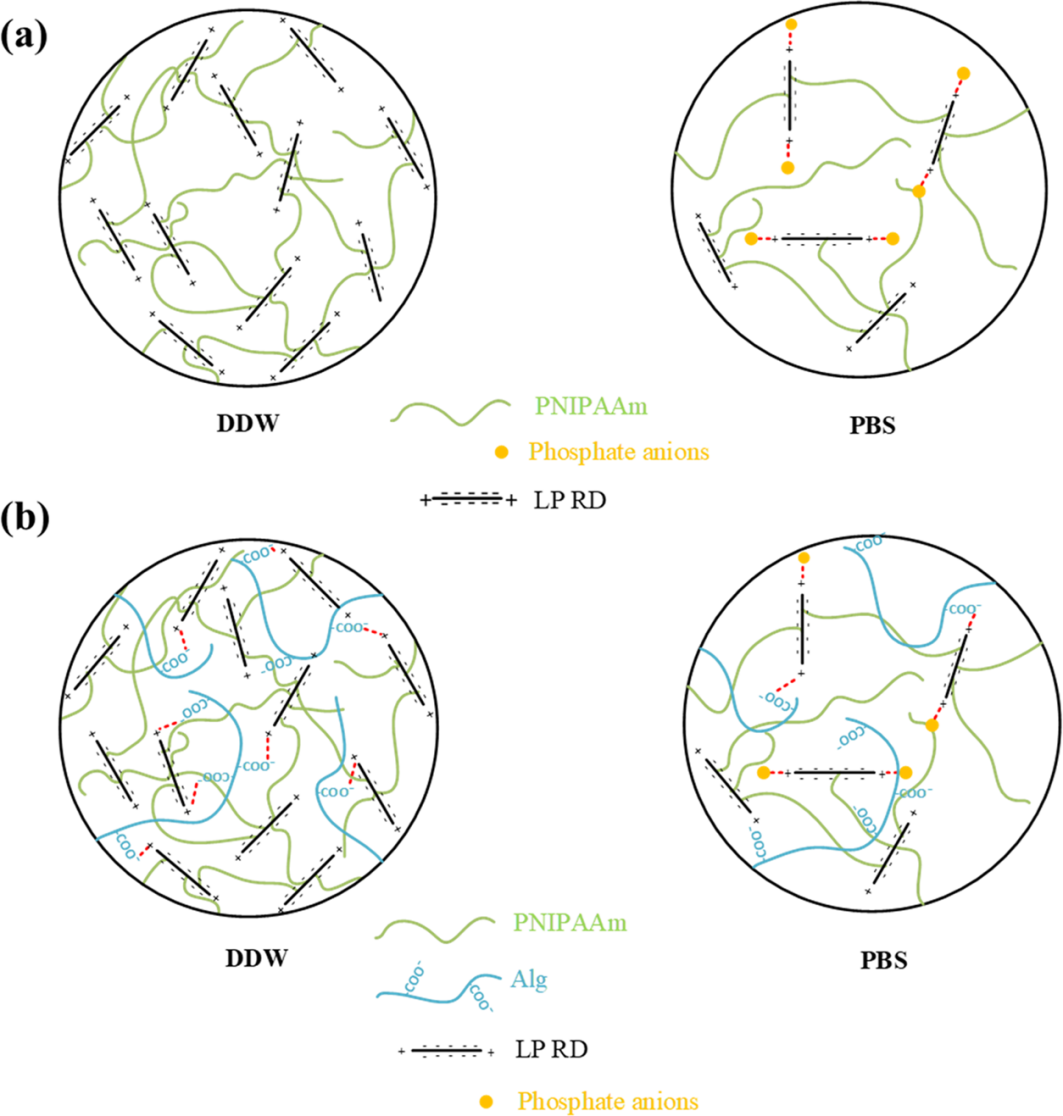
Schematic Representation of Possible Intermolecular Interactions between Laponite Layers and Phosphate/Carboxylate Anions for PNIPAAm (a) and Alg/PNIPAAm (b) Hydrogels Crosslinked with LP RD

Figure 1 and Scheme 2 show compressive Young’s moduli (E), compressive stress–strain curves, and possible intermolecular interactions at 25 and 37 °C in pH 5.5 DDW and pH 7.4 PBS of PNIPAAm and Alg/PNIPAAm hydrogels crosslinked with BIS and LP RD. It is seen that in the case of pH 5.5 DDW at 25 °C, all the hybrid PNIPAAm hydrogels exhibit much lower compressive Young’s moduli than the ones at 37 °C due to their swelling temperatures being below the VPTT of PNIPAAm chains. Although compressive Young’s moduli in PBS and DDW at 37 °C for PNIPAAm and Alg/PNIPAAm hydrogels crosslinked with BIS are higher than the ones in DDW at 25 °C, they exhibit nearly the same values. This means that the presence of phosphate anions in PBS solution does not change the hydrophilic/hydrophobic balance between the amide and isopropyl groups and the alginate chains, in the case of the B-series hydrogels (Scheme 2), whereas E moduli of LP RD-crosslinked PNIPAAm and its hybrid hydrogels in PBS at 37 °C are lower than those of both B-series in PBS and DDW and L-series in DDW at 37 °C. The main reason for this difference may be the electrostatic interactions between the phosphate anions in pH 7.4 PBS and the positively charged ends of the disc-shaped laponite layers. These interactions also contribute to the compressive strengths of L-series hydrogels, which are more swollen than their BIS-crosslinked counterparts.

It is known that the faces of laponite RD layers in water carry negative charges at pH < 9, while their edges are positively charged. Its result is that the laponite dispersions under these conditions organize into “house-of-card” structures through face-to-edge attractions and exhibit higher elastic moduli.^43−45^ The composition of both the hydrogels containing LP RD layers as multifunctional crosslinkers and the swelling medium could affect their distribution.

The electrostatic interactions of carboxylate anions on Alg chains with positive charges on the edges of laponite layers are dynamics, endowing the energy dissipation mode during compressive stress and resulting in the formation of more flexible and strengthened hydrogels compared to PNIPAAm under the same conditions (Figure 1b). Another reason why the E moduli of the L-series hydrogels in PBS are lower than those of DDW is the attractive forces between phosphate ions and positively charged ends of laponite layers. As a result of these electrostatic interactions, the negatively charged surfaces of the laponite layers will repel each other more, and as the degrees of swelling increase, the E moduli decrease.

**Figure 3:**
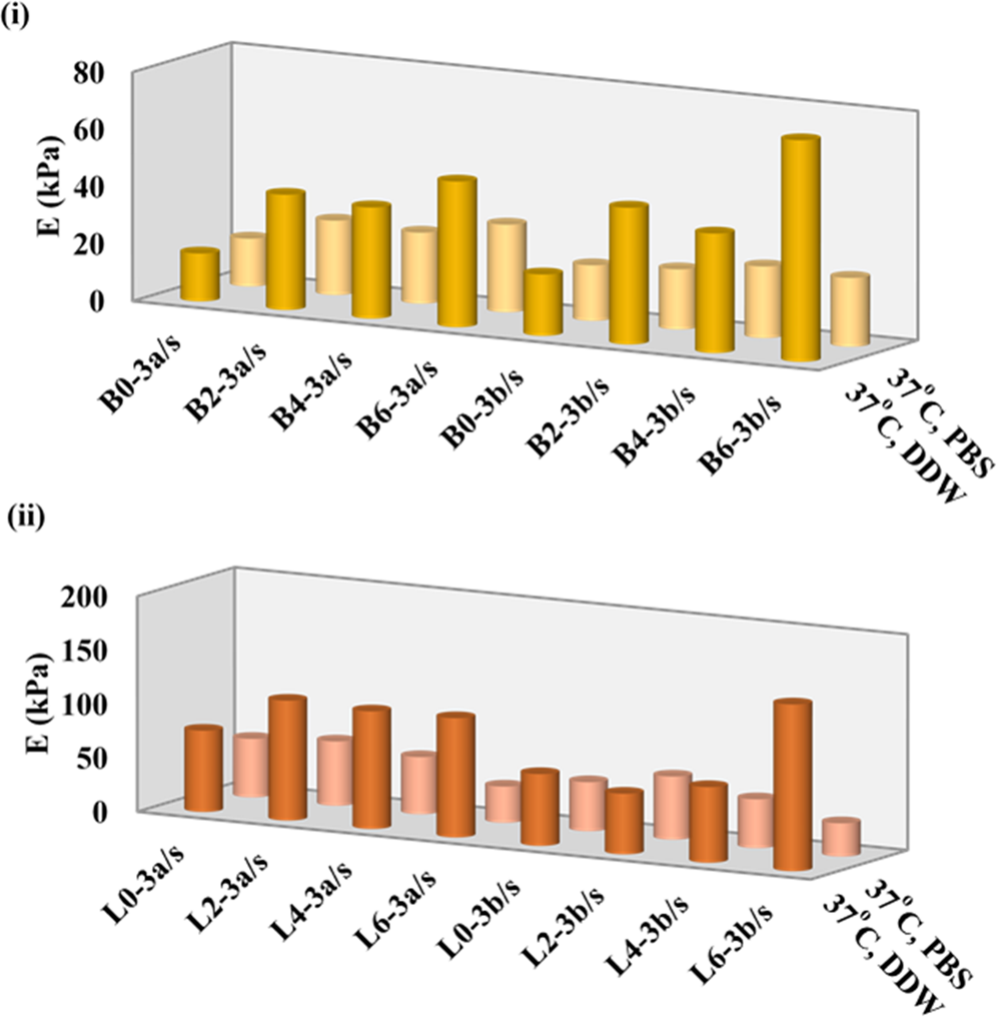
Compressive Young’s moduli at 37 °C in pH 5.5 DDW and pH 7.4 PBS for B-series (i) and L-series (ii) PNIPAAm hydrogels crosslinked with two different concentrations of Ce^3+^ ions.

The E moduli before and after crosslinking of B0/B2/B4/B6 and L0/L2/L4/L6 hydrogels with an external gelation method using Ce^3+^ ions in aqueous solutions of cerium(III) nitrate hexahydrate are given in Figure 3.

As for the results in Figure 1a, compressive Young’s moduli in PBS and DDW at 37 °C for single networks crosslinked with BIS molecules and LP RD layers in the absence of Ce^3+^ ions strongly decrease with increasing Alg content due to the hydrophilic nature of alginate chains. On the other hand, the change in the compression moduli of the double networks formed by ionotropic gelation between COO^–^ groups and Ce^3+^ ions exhibits a completely reverse order with regard to their corresponding single networks (Figure 3). For the two crosslinkers and two different Ce^3+^ concentrations, the compression moduli in DDW increase with increasing alginate content in the feed, since the carboxylate anions on the alginate chains act as crosslinking points in the presence of Ce^3+^ ions. Also, the E moduli of L2/L4/L6 in DDW are higher than those of B2/B4/B6 because of their increasing swelling degrees resulting with increased ionic gelation between COO^–^ and Ce^3+^ ions. Furthermore, the presence of NO^3–^ ions along with Ce^3+^ changes the dominance of hydrophobic forces in DDW at 37 °C in favor of hydrophilic interactions, whereas all E moduli in PBS are smaller than those in DDW. The reason for the latter was the phosphate ions in PBS solution, which were a typical buffering system used for biological agents, interacting with Ce^3+^ ions to form cerium orthophosphate, CePO_4_.^46,47^ It is known that cerium in aqueous solution may exist in two valence states, Ce^3+^ and Ce^4+^, depending on the conditions of the external environment, while the presence of PO_4_^3–^ ions in the buffer solution affects the redox cycle between Ce^3+^ and Ce^4+^.^48,49^

For the first time, Mino and Kaiserman suggested that cerium ions might form a redox pair in the presence of a reducing agent.^50^ In this study, we have also assumed that Ce^4+^ and Alg would form a redox system. Ce^4+^, which is one of the components of the redox pair, behaves as an oxidizing agent, while alginate, which is a member of polysaccharides, can act as a reducing component. After the complex formation step between hydroxyl groups on G and M units of alginate chains and Ce^4+^, active centers (i.e., free radicals) and Ce^3+^ ions are produced.^51,52^

**Figure 4:**
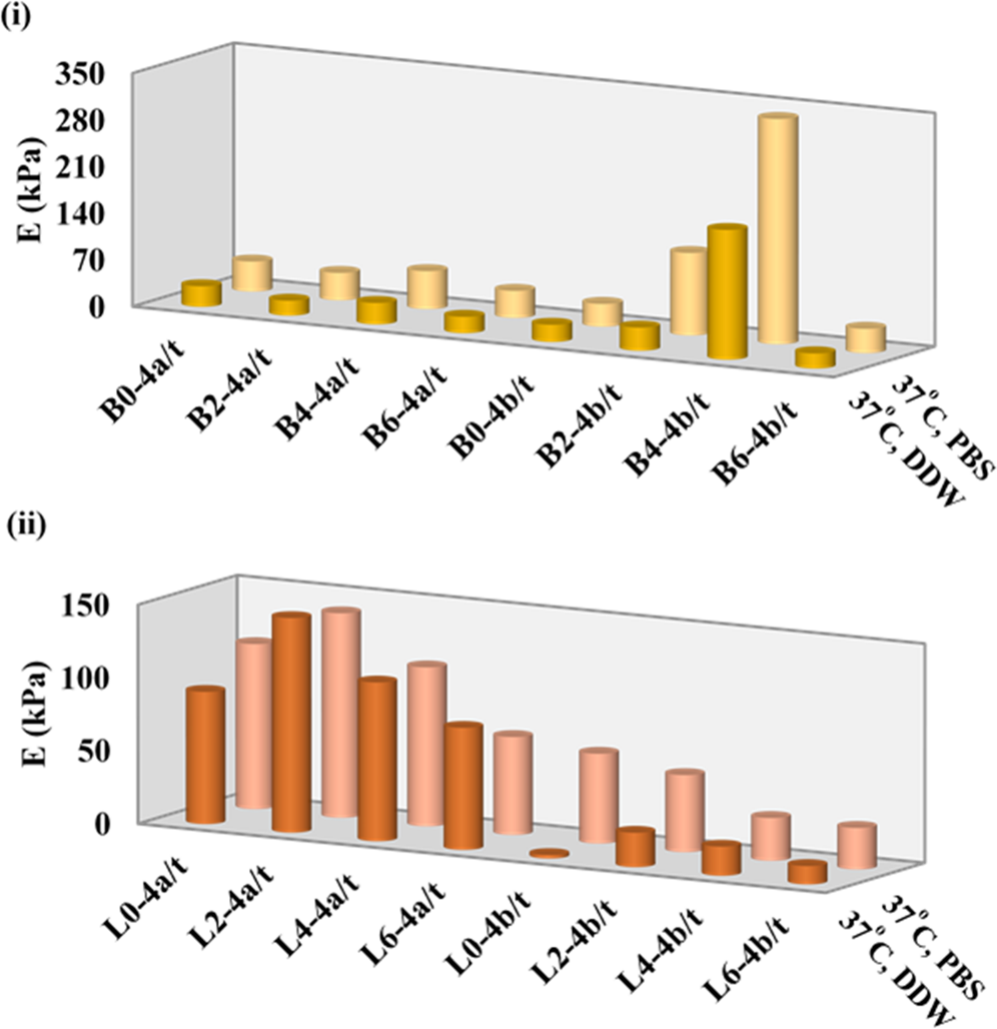
Compressive Young’s moduli at 37 °C in pH 5.5 DDW and pH 7.4 PBS for (i) B-series and (ii) L-series PNIPAAm hydrogels that reacted/crosslinked with two different concentrations of Ce^4+^ ions.

In this study, furthermore, the aqueous solutions of (NH_4_)_2_Ce(NO_3_)_6_ with two different concentrations were injected into glass tubes, including PNIPAAm and Alg/PNIPAAm hydrogels just after their polymerization periods without purification. The aim was to produce free radicals onto alginate and/or PNIPAAm chains for the formation of grafts and/or mutual termination since NIPAAm as a monomer would be present in the unpurified PNIPAAm and Alg/PNIPAAm hydrogels. According to the mechanism explained above, the decomposition of the complex between redox pairs produces Ce^3+^ ions and free radicals. In the following step, we have assumed that these Ce^3+^ ions could interact with G and/or M units of the Alg chains to facilitate ionic gelation and improve antibacterial activity. Figure 4 shows the E moduli in PBS and DDW at 37 °C for L0/L2/L4/L6 and B0/B2/B4/B6 hydrogels crosslinked using cerium(IV) ammonium nitrate, which acts as both ionic and covalent crosslinkers, to form double networks. When E moduli in Figure 3 of the hydrogels ionically gelated with Ce^3+^ ions are compared with the ones in Figure 4, it can be said that Ce^4+^ ions behave as a covalent crosslinker rather than an ionic one because the changes in E moduli are independent of the swelling solutions.

**Figure 5:**
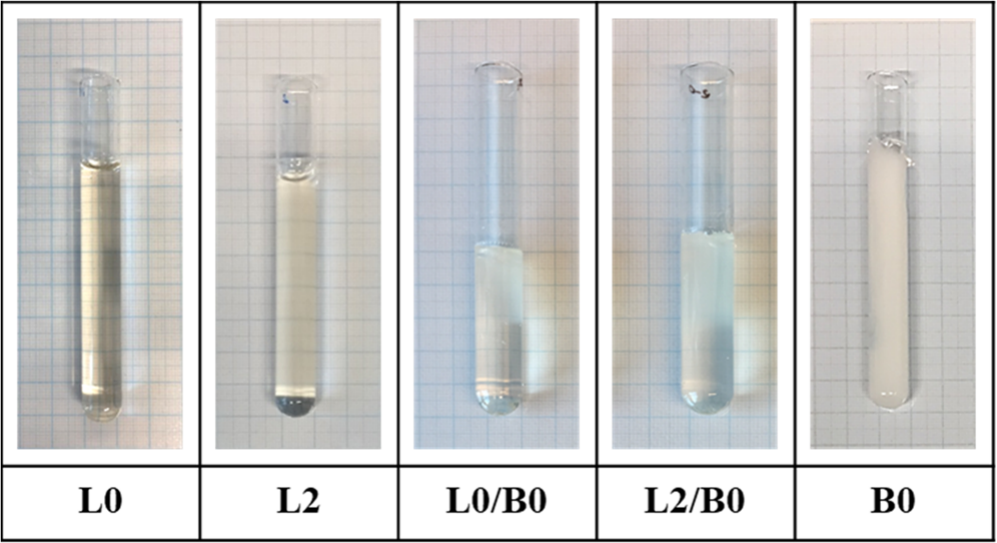
For the single and double networks of PNIPAAm (L0 and B0) and Alg/PNIPAAm (L2), digital photos of the appearances just after covalent crosslinking and before external gelation with Ce^3+^ ions.

The triple-network design in this study combines inorganic nanoparticles/cations with the synthetic and natural polymeric materials, to construct thermosensitive/antibacterial/hydrophilic/biodegradable hydrogels, which include nano-/macrostructures. Compressive Young’s moduli at 37 °C in pH 7.4 PBS for the double and triple networks formed in the absence and presence of Ce^3+^ are given in Table 1. They were obtained by starting from both single PNIPAAm hydrogels (B0 and L0) and LP RD-crosslinked PNIPAAm hybrid hydrogels containing 2.0 mol % Alg (L2). Figure 5 shows their appearances just after network formations.

**Table 1:** Compressive Young’s Moduli and Inhibition Zones at 37 °C in pH 7.4 PBS of Single (L0, B0, and L2) and Double Networks (L0/B0 and L2/B0) for PNIPAAm and Alg/PNIPAAm Hydrogels Before and After External Gelation with Ce^3+^ Ions

sample	*E*_37,PBS_ (kPa)	inhibition zone^a^ (mm^2^)	sample	*E*_37,PBS_ (kPa)	inhibition zone^a^ (mm^2^)
L0	88.9 ± 26.9	347	L0-3a/s	54.3 ± 1.6	386
L0/B0	12.2 ± 5.5	309	L0/B0-3a/s	122.3 ± 70.6	191
B0	169.0 ± 25.6	213	B0-3a/s	16.7 ±5.5	413
L2/B0	63.8 ± 30.4	279	L2/B0-3a/s	92.2 ± 45.8	178
L2	51.7 ± 33.3	397	L2-3a/s	59.7 ± 1.4	398

a All the disks were loaded with GS before inhibition zone tests.

**Figure 6:**
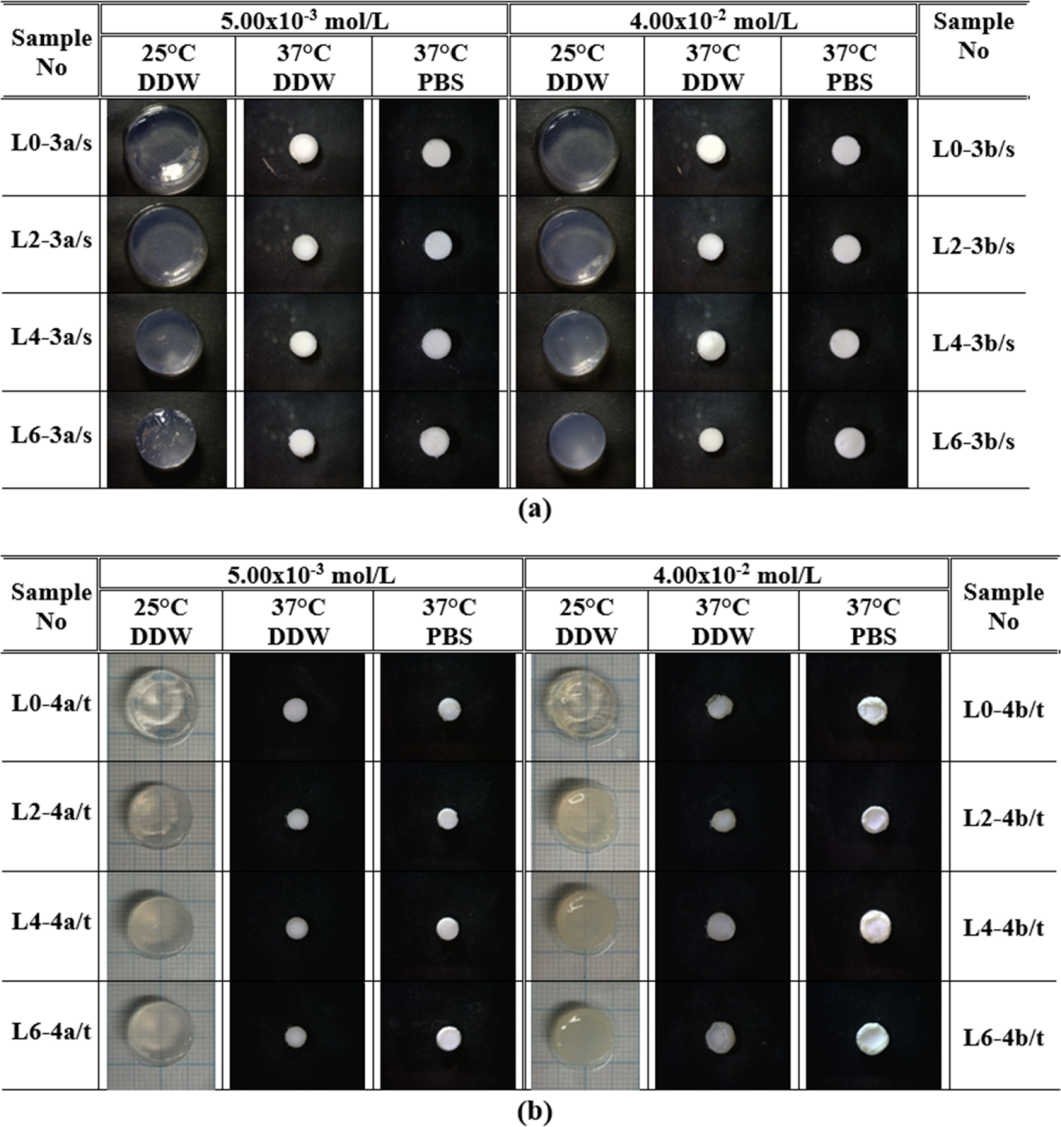
Digital photos at 25 and 37 °C in DDW and PBS of double networks prepared by external and internal gelations of PNIPAAm and Alg/PNIPAAm-LP RD hydrogels, using the aqueous solutions of cerium(III) nitrate hexahydrate (a) and cerium(IV) ammonium nitrate salts (b).

**Figure 7:**
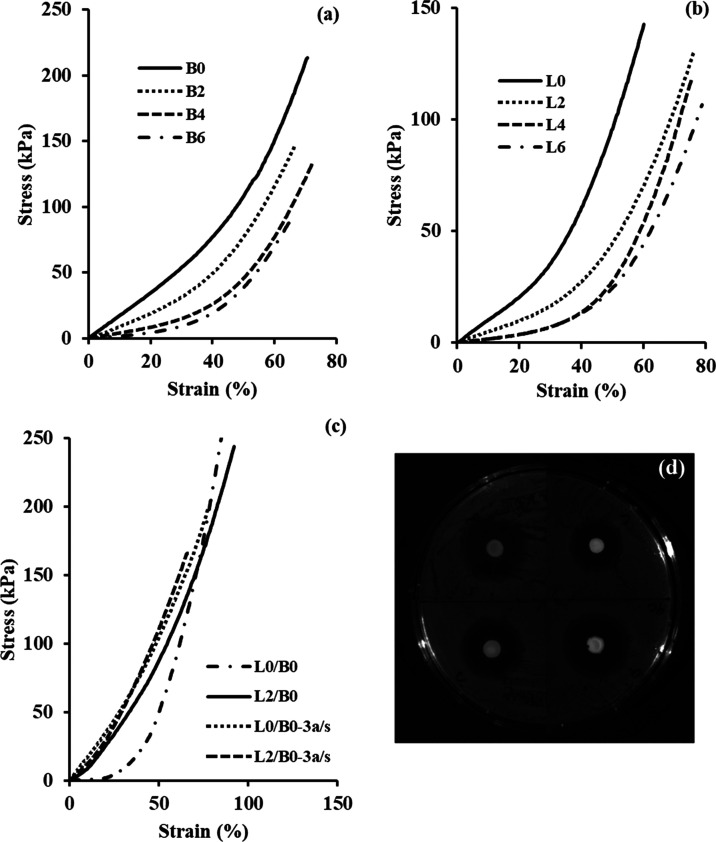
Compressive stress–strain curves of (a, b) PNIPAAm and Alg/PNIPAAm (B0-B6 and L0-L6) hydrogels, (c) L0/B0 and L2/B0 double networks, and their triple networks after external gelation with Ce^3+^ ions, and (d) antibacterial activities of GS-loaded L0/B0 and L2/B0 double networks and L0/B0-3a/s, and L2/B0-3a/s triple networks against *E. coli* bacteria. All the tests were performed at 37 °C in pH 7.4 PBS.

Thermosensitive nature that gives an advantage for drug release at body temperature and transparency for the ones synthesized at low temperatures (*T* < 25 °C) of PNIPAAm-based hydrogels increase their possibilities to be used as wound dressings. Despite these two advantages, that can also be modified and improved by structural arrangements, PNIPAAm hydrogels crosslinked covalently with BIS molecules have mainly two drawbacks: poor mechanical property and nonbiodegradability. To dissipate cracking energy, impart antibacterial property, improve mechanical strength, and increase water absorption capacity and transparency at room temperature, PNIPAAm-based/Alg-supported hybrid hydrogels were modified using two types of ionic crosslinkers, i.e., LP RD and cerium ions, instead of BIS. As seen form Figures 5 and 6, the increase in the transparencies of the double networks of PNIPAAm hydrogels before and after ionic gelation with Ce^3+^ ions at 25 °C originates from Alg chains and LP RD layers used as a hydrophilic component and ionic multicrosslinker, respectively. PNIPAAm hydrogels crosslinked with high content of BIS and prepared at 25 °C are opaque at just after polymerization and in swelling state. It is because, the PNIPAAm chains, exhibiting lower critical solution temperature (LCST) at around 32 °C, aggregate due to the exothermic nature of polymerization. This effect combined with high BIS concentration results in the formation of a heterogeneous microstructure. The source of the structural homogeneity (optical transparency) is the laponite layers, bearing many crosslinking points on each of them, instead of tetra-functional BIS molecules as crosslinker. As the lengths of PNIPAAm chains between crosslinking points increased, the number of laponite layers desreases that result in the formation of homogeneous microstructure.^13,16^ In addition, Alg chains used as natural component to prepare LP RD crosslinked-Alg/PNIPAAm hybrid hydrogels (L-series) support transpareny due to its ability to hydrate. Furthermore, it was expected that the presence of Alg chains in the double networks, i.e., L2/B0, should have increased the hardness after ionotropic gelations due to electrostatic interactions between carboxyl groups of Alg chains and Ce^3+^ ions (Table 1). However, the increase in the intermolecular interactions resulted in the enhanced compressive strength of double/triple networks but reduced diffusion of GS molecules and Ce^3+^ ions (Figure 7).

### Qualitative Antibacterial Activity Tests

In this study, we have proposed double and triple networks composed of PNIPAAm, alginate chains, and cerium ions with tunable mechanical performance and antibacterial activity. The combination of these materials overcame both the brittleness of alginate and low mechanical strength of PNIPAAm, while they were also supported by ionic crosslinks with Ce^3+^/Ce^4+^ ions.

The antibacterial activities against the Gram-negative bacterium *E. coli* of PNIPAAm and Alg/PNIPAAm hydrogels, which include Ce^3+^/Ce^4+^ ions, LP RD nanolayers, and BIS molecules, were studied by a disk diffusion method. The results of these double and triple networks were compared with those of both antibiotic drug GS-loaded disks and Ce^3+^/Ce^4+^ ion-impregnated paper disks (control test).

**Figure 8:**
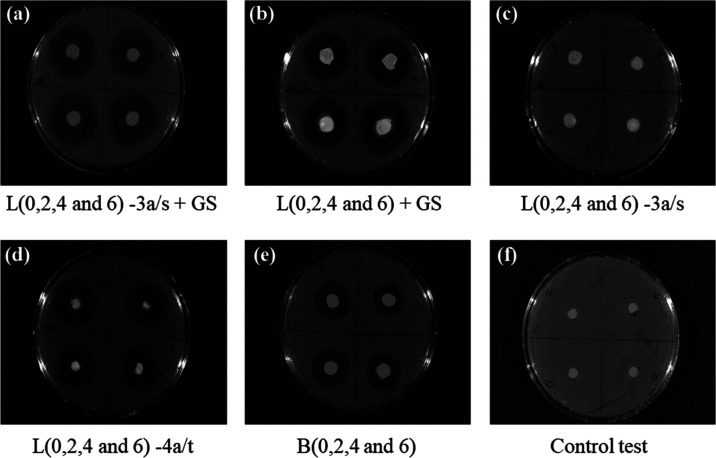
Photos of qualitative antibacterial tests against Gram-negative *E. coli* of PNIPAAm and Alg/PNIPAAm hydrogels: (a) crosslinked with LP RD and loaded by GS + Ce^3+^; (b) crosslinked with LP RD and GS-loaded; (c) crosslinked with LP RD and loaded by Ce^3+^; (d) crosslinked with LP RD and loaded by GS + Ce^4+^; (e) crosslinked with BIS and GS-loaded; and (f) control tests for two different concentrations of both Ce^3+^ (a, b) and Ce^4+^ (c, d) impregnated onto paper disks.

Table 2 and Figure 8 summarize the photos and numerical values of inhibition zone areas as a indication of antibacterial properties against *E.coli* of PNIPAAm and Alg/PNIPAAm hydrogel disks containing Ce^3+^/Ce^4+^ and GS as antibacterial agents. According to the results obtained, the inhibition zone areas of PNIPAAm hydrogels containing both Ce^3+^/Ce^4+^ and GS molecules, i.e., B0-3/s + GS, B0-4/t + GS, L0-3/s + GS, and L0-4/t + GS, were larger than the ones of B0 + GS and L0 + GS. The reason for the difference between them could be the antibacterial effect of Ce^3+^/Ce^4+^ ions, similar to the case of silver sulfodiazine–cerium nitrate pair used in deep burns.

**Table 2:** Inhibition Zones Against *E. Coli* Bacteria of PNIPAAm and Alg/PNIPAAm Hydrogel Disks Crosslinked with Ce^3+^/Ce^4+^ and Loaded GS

sample	inhibition zone (mm^2^)	sample	inhibition zone (mm^2^)	sample	inhibition zone (mm^2^)	sample	inhibition zone (mm^2^)	sample	inhibition zone (mm^2^)
B0 + GS	213	B0-3a/s + GS	413	B0-3b/s + GS	313	B0-4a/t + GS	313	B0-4b/t + GS	240
B2 + GS	340	B2-3a/s + GS	309	B2-3b/s + GS	302	B2-4a/t + GS	316	B2-4b/t + GS	248
B4 + GS	368	B4-3a/s + GS	337	B4-3b/s + GS	265	B4-4a/t + GS	301	B4-4b/t + GS	212
B6 + GS	425	B6-3a/s + GS	279	B6-3b/s + GS	256	B6-4a/t + GS	360	B6-4b/t + GS	350
L0 + GS	347	L0-3a/s + GS	386	L0-3b/s + GS	385	L0-4a/t + GS	413	L0-4b/t + GS	433
L2 + GS	397	L2-3a/s + GS	398	L2-3b/s + GS	421	L2-4a/t + GS	396	L2-4b/t + GS	358
L4 + GS	425	L4-3a/s + GS	428	L4-3b/s + GS	491	L4-4a/t + GS	326	L4-4b/t + GS	345
L6 + GS	440	L6-3a/s + GS	540	L6-3b/s + GS	588	L6-4a/t + GS	307	L6-4b/t + GS	321

The results obtained for control tests on Ce^3+^/Ce^4+^-loaded paper disks indicate the absence of an antibacterial effect, while the hydrophilic nature of PNIPAAm and hybrid PNIPAAm disks has a positive effect on the diffusion of cerium ions from the hydrogel disks along with GS molecules (Figure 8).

Zone areas of only GS-loaded hybrid hydrogels increase due to increasing hydrophilicity with an increase in the amount of alginate chains in Alg/PNIPAAm hydrogels (Table 2 and Figure 8).

On the other hand, it is seen that for the GS-loaded B0/B2/B4/B6 hydrogels including two different concentrations of Ce^3+^ ions, inhibition zone areas decrease with increasing Alg content, due to external gelation with COO^–^ ions on alginate chains, while in the cases of GS-loaded L0/L2/L4/L6 hydrogels, they exhibit a completely reverse order as to the ones crosslinked with BIS due to the positive contributions to the release rates of cerium ions of hydrophilic LP RD layers, that is, zone areas increase with the concentrations of alginate chains and cerium ions.

It is known that the growth of grafts onto the polysaccharide backbones is possible, which can be used as activators like chitosan and alginate chains, using Ce^4+^ ions as an initiator similar to KPS by grafting. Pourjavadi et al. reported the preparation of hybrid hydrogels crosslinked and initiated with BIS and ammonium persulfate (APS), respectively, in aqueous medium based on Alg chains, kaolin 1:1 layers, and polyacrylic acid (PAA) grafts. The interactions between reactive hydroxyl groups of silicate layers and carboxylate groups on Alg and PAA chains were characterized by FTIR spectra, indicating the formation of ester bonds.^53^ Trivedi et al. studied the effects of the reaction parameters on the graft polymerizations of ethylacrylate onto the backbones of alginate chains, using a Alg–Ce^4+^ redox pair, while Singh et al. synthesized the PAAm grafts onto the alginate backbones using Ce^4+^ (i.e., cerium(IV) ammonium nitrate).^54,55^

According to the polymerization mechanisms presented in these studies, the first step during the free radical graft copolymerizations of acrylate and amide monomers onto Alg chains is the radical formation facilitated by the redox reaction between their reactive hydroxyl groups and Ce^4+^ ions, after the disruption of the [Alg-Ce^4+^] equilibrium complex. Furthermore, in some cases, Ce^4+^ ions are used to terminate the growing polymer chains. Ce^4+^ here was used to observe the possibility of the radical and PNIPAAm graft formations onto Alg backbones by redox reactions and the antibacterial activities of Ce^4+^ ions or the contributions of Ce^3+^ ions produced during redox reactions. In fact, we assume that PNIPAAm grafts initiated onto the alginate backbones and/or free radicals formed by Ce^4+^ ions and reactive hydroxyl groups behave as a bridge between Alg chains and crosslinked PNIPAAm chains (Scheme 1). Figure 6 shows that the disk diameters at two temperatures in two different solutions of double networks of Alg/PNIPAAm hybrid hydrogels prepared using Ce^4+^ are smaller than those of the ones including Ce^3+^ ions. The formation of both Ce^4+^-induced covalent bonding and Ce^3+^-containing ionic gelation results in a double network structure because cerium(IV) ammonium nitrate solution was injected around the unpurified cylindrical Alg/PNIPAAm hybrid hydrogels crosslinked with BIS and LP RD. The disk diameters in the case of Ce^4+^ ions therefore can be smaller. The inhibition zones of double networks B2/B4/B6-4/t + GS and L2/L4/L6-4/t + GS also support these assumptions (Table 2). With increasing cerium(IV) ammonium nitrate concentration (from 5.00 × 10^–3^ to 4.00 × 10^–2^ mol/L) and alginate content (from 2.0 to 6.0%), it is seen that antibacterial activities decrease. Furthermore, the crosslinker type also affects the inhibition zone areas. For the same conditions, the double networks crosslinked with LP RD have wider areas, decreasing in a regular order with increasing alginate concentration, compared to the ones crosslinked with BIS due to the hydrophilic nature of laponite layers.

Triple networks were prepared by external gelation using Ce^3+^ ions after the syntheses of B0/L0 and B0/L2 double networks by starting from the single networks B0, L0, and L2 (Figure 5). Table 1 includes the inhibition zone areas of single, double, and triple networks containing both GS molecules and Ce^3+^ ions as antibacterial agents. It is seen that the antibacterial activities of GS-loaded double and GS + Ce^3+^-loaded triple networks are much lower than those of their single and double corresponds. This may be because increased structural complexity reduces the diffusion rate of the antibacterial agents.

## Conclusions

Single, double, and triple networks synthesized in the current study and composed of thermosensitive/biocompatible PNIPAAm polymers, hydrophilic/biodegradable alginate chains, and hydrophilic/biocompatible laponite layers are able to sense physiological temperature, control the diffusion rate of the antibacterial agents, GS molecules and cerium ions, and have a usage potential as wound dressings. Our results show that the sizes of inhibition zones and the values of E moduli change with (i) the hydrogel composition (PNIPAAm and Alg/PNIPAAm structures), (ii) the type of crosslinking (external gelation, multiple ionic, and divinyl-covalent bondings), (iii) the valence and concentration of cerium ions (two different initial concentrations of Ce^3+^ and Ce^4+^ ions), and (iv) the presence and amount of Alg chains. In addition, the formation of CePO_4_ in the presence of phosphate ions under physiological conditions also affects their mechanical and antibacterial properties by reducing the redox cycle between Ce^3+^ and Ce^4+^ ions. The lower values of compressive Young’s moduli in pH 7.4 PBS of the double networks of Alg/PNIPAAm hybrids containing cerium ions than those of the ones in pH 5.5 DDW also support these interactions. Due to the absence of phosphate ions in wound dressing applications on the skin, it can be expected that the antibacterial activities on *E. Coli* of the materials synthesized in the current study would be higher.
